# Carbon Nanotubes: A Summary of Beneficial and Dangerous Aspects of an Increasingly Popular Group of Nanomaterials

**DOI:** 10.3389/fonc.2021.693814

**Published:** 2021-07-27

**Authors:** Chengke Zhang, Licun Wu, Marc de Perrot, Xiaogang Zhao

**Affiliations:** ^1^Department of Thoracic Surgery, The Second Hospital, Cheeloo College of Medicine, Shandong University, Jinan, China; ^2^Key Laboratory of Thoracic Cancer, Cheeloo College of Medicine, Shandong University, Jinan, China; ^3^Latner Thoracic Surgery Research Laboratories and Division of Thoracic Surgery, Toronto General Hospital, University Health Network, University of Toronto, Toronto, ON, Canada; ^4^Department Immunology, University of Toronto, Toronto, ON, Canada

**Keywords:** carbon nanotubes, malignant mesothelioma, carcinogen, nanomaterials, asbestos

## Abstract

Carbon nanotubes (CNTs) are nanomaterials with broad applications that are produced on a large scale. Animal experiments have shown that exposure to CNTs, especially one type of multi-walled carbon nanotube, MWCNT-7, can lead to malignant transformation. CNTs have characteristics similar to asbestos (size, shape, and biopersistence) and use the same molecular mechanisms and signaling pathways as those involved in asbestos tumorigenesis. Here, a comprehensive review of the characteristics of carbon nanotubes is provided, as well as insights that may assist in the design and production of safer nanomaterials to limit the hazards of currently used CNTs.

## Introduction

Malignant mesothelioma (MM) is an aggressive neoplasm with poor prognosis that predominantly occurs in the mesothelial lining cells of the pleura and is hence termed malignant pleural mesothelioma (MPM) (1, 2). The association between MPM and asbestos exposure was first identified in 1956 in Cape Province, South Africa, and was further confirmed by a multitude of epidemiological studies (3). The efficacy of current therapies for MPM is very limited; thus, the MPM diagnosis almost invariably is a fatal one. Most cases of MPM can be linked to asbestos exposure in the workplace. The latency period of MPM is long, ranging from 13 to 70 years after first asbestos exposure (4). Even after the introduction of asbestos bans, the mesothelioma incidence rates continue to remain high or even rise (5).

Carbon nanotubes (CNTs) were first observed by Iijima in 1991 and are characterized by their nanosized hollow tube-shaped structures (6). Based on the number of layers, CNTs can be classified as either single-walled carbon nanotubes (SWCNTs) or multi-walled carbon nanotubes (MWCNTs). Both SWCNTs and MWCNTs are popular nanomaterials for commercial applications and widely used in fuel cell designs (7), photovoltaics (8), and biomedicine (9). The unique properties of nanomaterials have been extensively studied in various fields, and for instance the incorporation of nanotechnology is expected to contribute to sustainable energy. According to the Nanotechnology Consumer Products Inventory created by the Woodrow Wilson International Center for Scholars and the Project on Emerging Nanotechnology, 1,833 nanotechnology-based consumer products have been commercially available to date (10). It is estimated that 20,000 tons of CNTs will be produced by 2022 based on data from the Market Research Report (11). There are at least 38 commercial products containing CNTs on the market (12).

CNTs pose a potential occupational health risk. CNTs are similar in size and shape to asbestos and have the same biopersistence, and several studies on CNTs have focused on the carcinogenicity of these compounds and studied granuloma formation, fibrosis, mesothelial proliferation, and mesothelioma-like growth (13–15). In this review, we will first summarize some of the applications of CNTs in nanomedicine. We will then review animal studies that analyzed the biological effects of the physicochemical characteristics of CNTs (including length, diameter, surface modification, rigidity, and curvature) on pleural mesothelial cells. Thereafter, we will discuss the underlying molecular mechanisms involved in the interaction between mesothelial cells and CNTs. Finally, we will underline the need for both public and governmental awareness of CNT-induced mesothelioma and the importance of regulatory mechanisms to limit the potential damage elicited by CNTs during manufacturing and disposal.

## Applications of CNT in Nanomedicine

CNTs represent a promising class of nanomaterials for medical application. Researchers have demonstrated that CNTs can be used for drug (16–19) and biomolecule transport (20), tumor imaging (21), and photothermal therapy (22).

Nanoparticles, such as CNTs, can actively or passively target malignant tumor cells. Passive targeting relies on the enhanced permeability retention (EPR) effects of cancerous tissue. It is well-established that the endothelium of tumor blood vessels is more penetrable than that of the “healthy” endothelium. These leaky vessels allow nanoparticles to penetrate into the tumor stroma. Absence of normal lymphatic drainage in tumors will also contribute to nanoparticle retention. Active targeting of malignant tumor cells by CNTs relies on tumor-targeting ligands such as folic acid (23), antibodies (24), or aptamers (25), all of which seem to enhance the intracellular uptake of nanoparticles.

### Anticancer Drug Carriers

As biological carriers, CNTs are able to transport cytotoxic drugs such as doxorubicin (17–19) and the prodrugs of cisplatin (23, 26) and paclitaxel (16) across the cellular membrane *via* endocytosis. CNTs may be used to deliver drugs to (targeted) cancer cells, potentially achieving better results than drugs without carrier. Targeted drug delivery has the potential to reduce systematic toxicity and increase the concentration of drug molecules in tumors. Moreover, the release of drugs from CNTs can be manipulated by changing the pH, potentially further improving therapeutic efficiency. Due to the acidic microenvironment presents in many solid tumors, CNT-drug complexes may be easily released (17).

Paclitaxel (PTX) can be attached to branched PEG-coated SWCNTs *via* a cleavable ester bond to obtain a water-soluble SWCNT-PTX conjugate (16). Because of its prolonged blood circulation time and enhanced uptake by tumors, the SWCNT-PTX conjugate seems to be more efficient than non-conjugated PTX in suppressing tumor growth *in vivo*. Additionally, aromatic molecules, such as doxorubicin, can be loaded onto the surface of PEGylated SWCNTs (17, 18) or pluronic-dispersed pristine MWCNTs (19) through supramolecular π-π stacking. It has been demonstrated that the complex of doxorubicin and SWCNT inhibits tumor growth to a greater extent than free doxorubicin *in vivo* (18). Another example of the utility of CNTs in drug delivery is the use of them as drug carriers for the transportation of platinum prodrugs (26). After being ingested by cancer cells through endocytosis, platinum prodrugs may then be reduced to active cisplatin and able to inhibit cancer cell replication.

Moreover, studies have found that CNTs can be used as transporters for siRNA therapeutics (20, 27). For instance, SWCNTs functionalized with positively charged groups carrying telomerase reverse transcriptase siRNA show tumor growth inhibition effects *in vivo* (27). Additionally, amino-functionalized MWCNTs that bind to toxic siRNA sequences can inhibit tumor growth and increase the survival rate of tumor-bearing mice (20), which demonstrates that chemically functionalized CNTs can be used as transporters of siRNA in cancer therapy.

### *In Vivo* Tumor Imaging Techniques

Because of their unique physical-chemical characteristics, including large surface areas, strong near-infrared (NIR) absorbance, and good photothermal performance, CNTs have a great biomedical potential.

#### Positron Emission Tomography (PET)/Single-Photon Emission Computed Tomography (SPECT)

Researchers have shown that SWCNTs can be also used as radio-imaging probes *in vivo* either by attaching radionuclides such as ^64^Cu, ^111^In, and ^125^I to their surfaces or filling radionucleotides into the center of the SWCNTs (28–30). The major advantage of these two approaches is that radionuclide-labeled SWCNTs can then enhance imaging sensitivity and penetration depth. A study using PET images demonstrated that PEGylated SWCNTs conjugated with ^64^Cu and the targeting peptide arginine-glycine-aspartate (RGD) efficiently targeted α_v_β_3_-positive tumors *in vivo* (28). Another study found that an anti-CD20 antibody linked to SWCNTs labeled with ^111^In could be used to target human Burkitt lymphoma *in vivo* (31). In addition to modifying the surfaces of SWCNTs, researchers have also filled SWCNTs with the radionuclide Na^125^I for SPECT/CT imaging (30).

#### Magnetic Resonance Imaging (MRI)

Contrast agents such as Gd-DTPA and iron oxide nanoparticles are commonly used in MRI to enhance the contrast between normal and abnormal tissues. Gd-DTPA and iron oxide nanoparticles shorten the T1 and T2 relaxation times, respectively, thereby changing the signal intensity on the images. Researchers have found that gadolinium chelates conjugated to MWCNTs are useful as a T_1_-weighted contrast agents (32, 33). In other studies, iron particles embedded into or loaded onto the surface of MWNCTs were used as T_2_-weighted MRI contrast agents (34, 35). In addition, non-covalent functionalization of MWCNTs with amphiphilic Gd^3+^ chelates creates both T_1_- and T_2_-weighted MRI contrast agents (36). Furthermore, ion-tagged SWCNTs conjugated with an Endoglin/CD105 antibody facilitated the detection of tumors *in vivo* using non-invasive MRI (37). This demonstrates that an ongoing integration of CNTs in MRI potentially assists in more efficient detection of solid tumors.

#### Photoacoustic Imaging

Photoacoustic imaging overcomes the limitations of traditional technologies based on photoacoustic effects because of its promising non-ionizing, non-invasive, and sensitive molecular imaging technology. In fact, it is suggested that photoacoustic imaging may assist cancer diagnosis and may provide treatment guidance (38). Apart from localizing tumors, photoacoustic imaging may also be used to visualize tumor vasculature/angiogenesis. Furthermore, SWCNTs conjugated with RGD peptides may also be employed as contrast agents (39).

#### Near-Infrared Photoluminescence

The near-infrared (NIR) photoluminescence of SWCNTs has made them a promising NIR fluorescent contrast agent in molecular imaging. SWCNTs, as the contrast agent, can detect emissions in the range of 1,100–1,400 nm and is able to achieve deep tissue penetration. Suspended by a phospholipid PEG, SWCNTs have relatively high quantum yield and biocompatibility. After functionalization with the RGD peptide, the use of SWCNTs allowed high-resolution imaging of the tumor vasculature of tumors (40). SWCNTs were also found to passively accumulate in tumors (EPR effect), and increased NIR fluorescence could be detected in the tumor region (41).

#### Raman Imaging

The inherent G-band of Raman spectra provides a tool for quality measurements of tumors and biological imaging with nanotubes (28). In one study, Raman spectroscopy was used to evaluate tumors in mice *in vivo* with RGD-targeted SWCNTs (42). In addition to single-color SWCNT Raman imaging, multicolor Raman imaging with SWCNTs was explored by manipulating different isotope compositions (43). Studies have demonstrated that surface-enhanced Raman scattering can be used to enhance detection sensitivity and reduce imaging time (44, 45).

### Multifunctional Nanoparticles

Multifunctional nanoparticles were developed through integrating different functional agents, such as specific targeting moieties, imaging and therapeutic agents, into a single nanocontruct (46, 47). One study showed that magnetofluorescent carbon quantum dots/doxorubicin could be conjugated to MWCNTs as multifunctional nanocomposites (48). The same researchers have discovered another multifunctional SWCNT nanocomposite for potential therapeutic use. They demonstrated that this nanocomposite simultaneously served as a doxorubicin carrier, an NIR photothermal heater, and a multimodal imaging probe (25). These nanocomposites have the potential to combine tumor imaging, photothermal therapy, and chemotherapy.

Despite their impressive potential in biomedical applications, CNTs may still possess asbestos-like toxicity as a consequence of their nanostructure and biopersistence. In the next section, we will discuss the potential of CNTs to induce mesothelioma-like pathology as noted in animal studies.

## Experimental Evidence of CNT-Induced Mesothelioma in Animal Models

Adverse effects of CNTs were studied in pulmonary models (49–51). They were able to induce oxidative stress/inflammation, fibrosis, granulomatous lesions, and respiratory tumors. CNTs were also shown to be capable of promoting angiogenesis and tumor invasiveness (52). CNT-induced testicular damage (53) and reproductive and developmental toxicity were also reported (54). The research related to CNT-induced mesothelioma tumorigenesis is limited to animal experiments (14, 55, 56). MWCNT-induced mesothelioma exhibits similar key pro-oncogenic molecular events as seen in human MPM (57).

### Fiber Pathogenicity Paradigm of CNTs

Both asbestos and CNTs have high aspect ratios. The fiber pathogenicity paradigm, in which the length, diameter, and biopersistence of fibers are identified as crucial toxicological characteristics, has been extended to include man-made, high-aspect-ratio nanomaterials such as CNTs (58, 59). The rigidity of nanofibers was proposed as another parameter to be added to the toxicological profile of CNTs (60). Critical reviews have summarized the similarities and differences in physicochemical properties of CNTs and asbestos. They include surface charge, hydrophilicity/hydrophobicity, impurities, tensile strength, and biopersistence (60, 61). Compared to asbestos, CNTs represent a diverse group of nanoparticles with varying physiochemical characteristics, including wall number, length, diameter, and surface modification. SWCNTs and MWCNTs have diameters ranging from nanometers to tens of nanometers and lengths ranging from several hundred nanometers to several millimeters. In addition, surface modification of CNTs alters surface chemistry and increases diversification, which eventually changes the protein-binding, cytotoxicity, and immune response related to CNTs *in vivo* (62, 63).

Absorption, biodistribution, metabolism, and excretion (ADME) of nanoparticles are expected to play a major role in the toxicology of nanomaterials. Unique physicochemical characteristics of CNTs (length, diameter, surface modification, and exposure routes) determine their ADME and biological effects (64). The pulmonary tract, skin, and gastrointestinal tract are the most common sites of exposure to CNTs. CNT-induced dermal toxicity resulting from localized dermal exposure was associated with the generation of free radicals, oxidative stress, and inflammation (65). Alternatively, it has been found in animal studies that CNTs entering the body *via* oral exposure reach the gastrointestinal tract as agglomerates and seem to be mostly excreted in feces without behavioral abnormality, change in body weight, or pathological abnormality of animals (66, 67).

Thin fibers with diameters less than or equal to 1 µm and long fibers once inhaled were found to easily penetrate the ciliated airways (61). The nanosized CNT particles are deposited in the alveolar space and then penetrate into the lung parenchyma and eventually reach the pleural space. The exact transition pathways have not yet been identified. Several hypotheses have been developed (68–70). Penetration of alveolar macrophages, the alveolar wall, and the visceral pleura by CNTs is a well-recognized phenomenon (70). Long fibers are supposed to have limited mobility and will thus remain in the interstitium of the lung or the pleura, whereas short fibers and particles may spread more widely. The biopersistence of CNTs is another important element of carcinogenicity. Some SWCNTs and MWCNTs can be degraded by enzymatic systems *in vitro* and by macrophages *in vivo*. CNT type, the length, surface modifications, impurities, and dispersion state contribute to the biopersistence of CNTs *in vitro* (71). Both biodegraded CNTs and their by-products do not show toxicity *in vitro* as well as *in vivo* (71). Retained long CNTs can induce an acute inflammatory response followed by influx of inflammatory granulocytes (68) and release of cytokines and oxidants by (pleural) macrophages called “frustrated phagocytosis”. This process results in continued inflammation, fibrosis, and genotoxic effects in nearby tissues. A similar mechanism is held responsible for the development of mesothelioma by asbestos.

Experimental studies of exposure routes of CNTs have shown that inhalation, intratracheal intrapulmonary spraying, intrapleural injection, and intraperitoneal injection may all result in mesothelioma development (55, 57, 72–77). Both asbestos and CNT fibers can be “aerosolized,” and inhalation of CNTs is considered the most frequent way of fiber exposure in humans.

In summary, MWCNTs represent a potential new carcinogen able to elicit MPM. Therefore, the risks of exposure to CNTs should be thoroughly evaluated and adequate preventive measures taken. The physicochemical properties of CNTs, exposure facts, and outcomes of research studies are summarized in **Supplementary Table 1**.

### Intraperitoneal or Intrascrotal Injection

The biological effects following injections of CNTs in the peritoneal cavity resemble similar injections in the pleural cavity (58). A pilot study in mice showed that inflammation and granuloma lesions on the peritoneal side of the diaphragm developed after intraperitoneal injection of two types of MWCNTs (MWCNT1 length (L)=13 µm, diameter (D)=84 nm, and MWCNT2 L=56 µm, D=165 nm) (14). Absence of mesothelioma growth in this study is ascribed to short and low-dose exposure. Another study showed that intraperitoneal administration of MWCNT-7 (L=less than 5 µm, D=100 nm) at a dose of 3 mg (corresponding to 1×10^9^ fibers)/mouse developed mesothelioma in 87.5% of p53 heterozygous mice after 84 days (55). The cumulative incidence of mesothelioma induced by MWCNT-7 was found to be related to the exposure dose (300 µg (1×10^8^ fibers)/mouse, 30 µg (1×10^7^ fibers)/mouse, and 3 µg (1×10^6^ fibers)/mouse) in p53 heterozygous mice (72). This research demonstrated that MWCNTs, specifically MWCNT-7, are capable of inducing mesothelioma.

Compared with the other two types of MWCNTs with similar lengths (less than 10 µm) and diameters of 150 nm and 2–20 nm (tangled), MWCNT-7 is more toxic and has a greater potential to develop inflammation and mesothelioma *in vivo*, most likely as a consequence of a small diameter and high crystallinity (73). With these characteristics, MWCNT-7 can penetrate mesothelial cells and induce frustrated phagocytosis. Furthermore, straight, acicular-shaped MWCNTs, such as MWCNT-7, are more likely to induce mesothelioma through intraperitoneal injection or intrascrotal injection (56, 74). Curved and bent MWCNTs show less toxic carcinogenic potency than their counterparts that are straight and needlelike (75).

In summary, the characteristics of CNTs are considered critical factors for their carcinogenesic potential. CNTs that are rigid, needlelike, and have a length of 5 µm and a diameter of 50 nm are more likely to induce mesothelioma growth.

### Airway Exposure

The most important route for human exposure to CNTs is the inhalational route, and pharyngeal or intratracheal installation is the preferred methods for CNT administration in animal studies. These studies require a sophisticated laboratory setting (78, 79).

Those MWCNTs that are able to reach the subpleural tissue after inhalation and penetration into the lung parenchyma set the foundation for MPM development (80). Previous studies have shown that MWCNTs could induce the development of subpleural fibrosis in mice (80) and dose-dependent mesothelial hyperplasia in the parietal pleura of rats (81). CNTs administered through pharyngeal aspiration are also able to reach subpleural tissues, translocate into the pleural cavity, and deposit in the parietal pleura (70, 82). MWCNTs retained in the parietal pleura may then cause pleural inflammation and development of (pre)-malignant lesions in a length-dependent manner (82).

Through airway exposure, MWCNTs are associated with pleural inflammation, fibrosis, and mesothelial proliferation in both the visceral and parietal pleura (83, 84). However, in one study, the MWCNT-induced inflammatory responses in the pleural cavity and hyperplastic visceral mesothelial proliferation could have been affected by the short experimental period (15). It was found in other research that SWCNTs with a length of 0.5 µm were able to induce similar inflammatory effects as seen with MWCNTs (85).

Both MWCNT-7 and MWCNT-N can induce mesothelioma growth within 1 year of exposure (76, 77). However, one study found that inhalation exposure to MWCNT-7 with a co-treatment of a known carcinogen, methylcholanthrene, induced mesothelioma, whereas mice exposed to MWCNT-7 alone did not develop mesothelioma (86). Other studies with 2 years of MWCNT-7 administration suggested that a higher quantity of fiber distribution in the pleura may be necessary for mesothelioma induction (70, 81), or that certain mouse models might be resistant to mesothelioma development (87).

In summary, fiber length plays an important role in the inflammatory induction of mesothelioma growth. The persistent CNTs that were able to reach the pleura after inhalation and lung penetration are considered the necessary elements for malignant transformation. Also in humans, the development of malignant mesothelioma is assumed to be the consequence of the inhalation of CNTs, which is followed by a long latency period before cancer is diagnosed.

### Intrapleural Injection

CNTs can also be administered through intrapleural injection (through the thoracic wall). Research has shown that length-dependent retention of CNTs in the pleural cavity is able to induce sustained inflammation, progressive fibrosis, and mesothelioma growth (57, 68, 88). The molecular mechanisms involved in mesothelioma development caused by CNT exposure are considered the same as in asbestos carcinogenesis (57). By investigating the experimental outcome of a series of nanoparticles with different sizes, the relationship between fiber length and pleural inflammation was determined, and results showed that the dimension threshold for pleural inflammogenicity was 5 µm for mice, and the resultant data could be extrapolated to the human situation (89).

In addition to length, the rigidity of CNTs is an important physicochemical determinant of toxicity. Rigidity is an indicator of fiber curliness and is characterized by the bending ratio and static bending persistence length. A bending ratio of 0.97 and a static bending persistence length of 1.08 are the threshold rigidity values for asbestos-like pathogenicity (90). Also, surface modification is an important factor affecting the biological effects of CNTs. For instance, MWCNTs with surface modification using ammonium-terminated tri(ethylene glycol) chains result in less granulomas on the mesothelial membrane than pristine and alkyl-functionalized MWCNTs with the same effective length (91), which suggests that surface modification can alleviate the negative effects of CNTs.

Research based on animal models suggests that mesothelioma development induced by MWCNT-7 may have a shorter latency period (55, 56, 77) and a higher incidence rate (87) than that by asbestos. Three reasons may explain these differences. First, MWCNT-7 fibers are more likely to cause DNA damage than asbestos fibers that are surrounded by vesicular membrane structures in cytoplasm after MWCNT-7 fibers or asbestos fibers crossing celluar membranes of mesothelial cells (73). Second, CNTs interact with biological components in physiological environment or cell culture media and are surrounded by a protein corona (92). The components in protein corana affect the toxicity of nanoparticles (92). For example, transferrin is specially contained in protein corona of MWCNT-7 but not asbestos (93, 94). Transferrin absorbed on surface MWCNT-7 results in mesothelial iron overloading, which causes possible carcinogenesis of mesothelial cell and high levels of oxidative stress. Third, the immunosuppressive microenvironment elicited by CNTs may be different from asbestos and contains more myeloid-derived suppressor cells (87).

Certain issues remain unknown and need to be further explored. For instance, it is not known whether CNTs administered by intravenous injection may eventually arrive in the pleura. Furthermore, the threshold dose of fibers deposited in the pleural cavity to induce MPM remains unclear. A previous study showed that only 10^3^ MWCNT-7 fibers deposited in the pleura were able to induce mesothelial hyperplasia. The dose was 10^6^-fold smaller than the dose required to induce lung cancer (70). It is not excluded that the amount of fibers able to reach the pleura may determine toxicity rather than the physicochemical features of CNTs (84).

**Figure 1:**
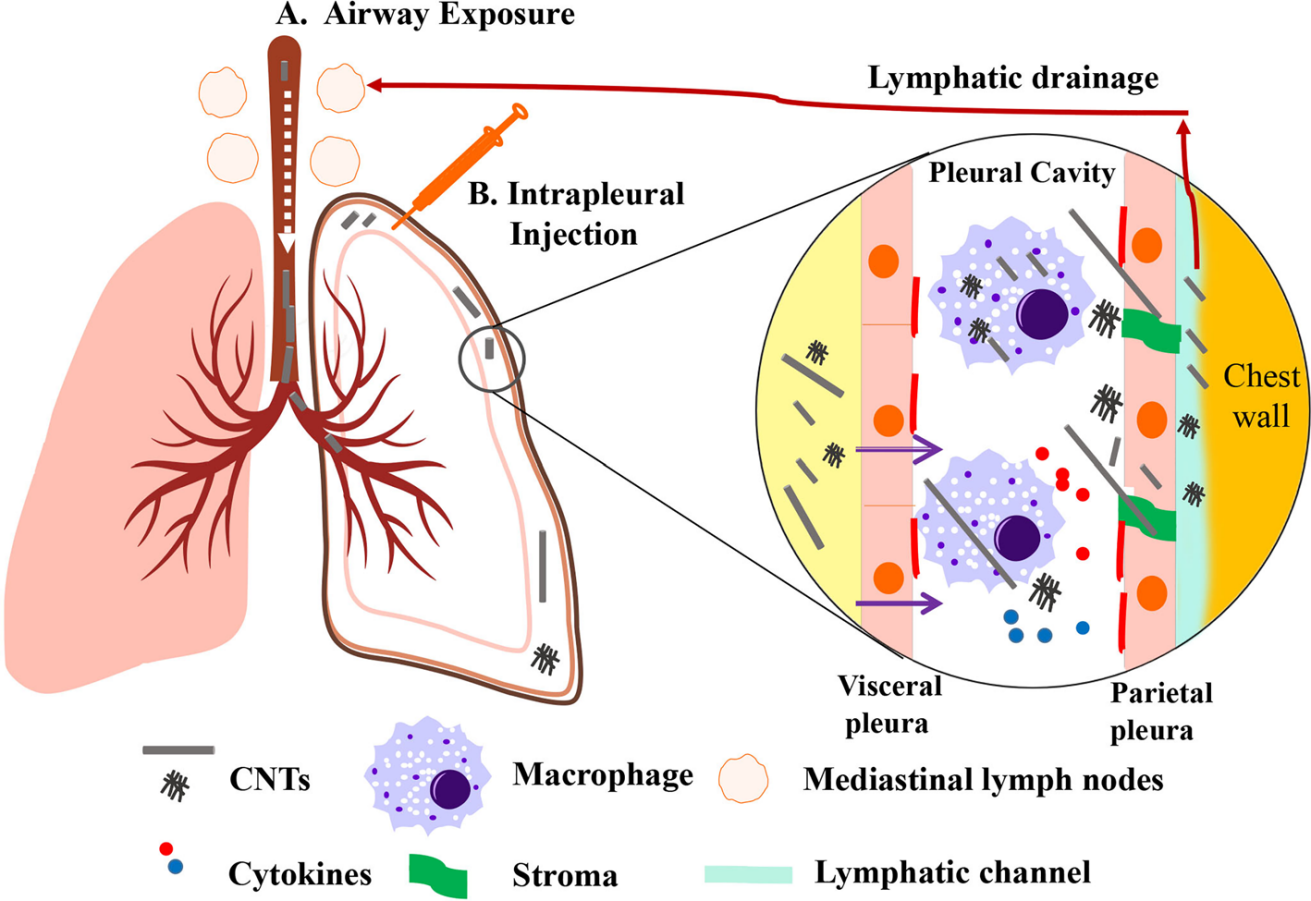
Representative scheme of CNT drainage and pathological lesions induced by CNTs. CNT administered through airway exposure then penetrates the pulmonary alveoli and visceral pleura into the pleural cavity **(A)**. CNT can also be administered to pleural cavity through intrapleural injection **(B)**. Small-sized CNT or CNT aggregates lead to mild pathological lesions due to its engulfment by macrophages and lymphatic drainage. Long, rigid, and needlelike CNTs deposited in pleural cavity fail to negotiate with the stroma. It can induce pro-inflammatory cytokines and oxidants released by macrophages due to frustrated phagocytosis. Long CNTs are located in fibrotic parietal pleura, while a few CNTs could pierce and penetrate into the parietal mesothelium. [Modified from (58)].

Taken together, inhaled CNTs are able to penetrate the airway epithelium, migrate through the lung parenchyma, and reach the pleura. Short or tangled CNTs seem to move rapidly and easily reach the pleural space to be released into the lymphatic system, whereas longer CNTs will move more slowly. Longer and needle-shaped CNTs are able to pierce mesothelial cells and macrophages, which results in frustrated phagocytosis with the consequent production of high levels of pro-inflammatory cytokines (**Figure 1**). All published studies have shown so far that MWCNT-7 is capable of inducing mesothelioma-like growth in animal models. Nevertheless, there is a lack of epidemiologic evidence that relates CNTs to MPM induction in humans.

## Mesothelial Cell Damage After CNT Exposure

Recent findings have shown that CNTs have the potential to initiate or develop mesothelioma growth. The transformation from healthy mesothelial cells to mesothelioma cells is marked by cell proliferation and invasion. Based on several investigations, CNTs exhibit features that correspond to the 10 key characteristics of carcinogens (95). For example, CNTs may enter cell nuclei and directly cause DNA damage to mesothelial cells. Furthermore, nanoparticles, including CNTs, can generate reactive oxygen species (ROS), the most significant mechanism of nanotoxicity (96), and activate a signaling cascade that may eventually result in cancer. Lastly, pro-inflammatory cytokines are released from macrophages due to CNT exposure *via* frustrated phagocytosis. Mesothelial cells are also damaged by other CNT-induced incidents, including immunosuppressive microenvironment and epigenetics (87, 88). All these conditions perturbed by CNTs may contribute to the transformation of healthy mesothelial cells to MPM cells.

The penetration of mesothelial cells by CNTs is considered one of the important first steps in carcinogenesis. Fiber-shaped materials such as asbestos and CNTs may directly disrupt the mesothelial cells. CNTs may enter the mesothelial cells through energy-dependent endocytosis and energy-independent piercing. MWCNT-7 invades the mesothelium through three pathways: clathrin-mediated endocytosis, caveolae-mediated endocytosis, and micropinocytosis (97). Additionally, thin MWCNTs with diameters of 50 nm can pierce the plasma and nuclear membranes of human mesothelial cells and remain in the cytoplasm without being covered by vesicular membrane structures (73).

One of the most studied mechanisms related to nanoparticle-induced toxicity is oxidative stress, which results from an imbalance between free radicals and antioxidants in cells. Oxidative stress induced by nanoparticles is able to cause cell toxicity and tissue or organ damage both *in vitro* and *in vivo* (96). Overproduction of ROS can activate pro-tumorigenic signaling, enhance cell survival and proliferation, and drive DNA damage and genetic instability (98). SWCNTs and MWCNTs can cause DNA damage and decrease cell viability by stimulating the generation of ROS in human mesothelial cells (99–102). In addition, chronic (low-dose) exposure of SWCNTs or MWCNT-7 to human mesothelial MeT5A cells can induce cell proliferation, migration, and invasion through ROS production (103).

CNTs are able to induce an immunosuppressive environment by recruiting immunosuppressive monocytic myeloid-derived suppressor cells and macrophages (87). Macrophages release pro-inflammatory cytokines such as IL-1β and TNFα *via* frustrated phagocytosis, which may lead to fiber-induced inflammation, mesothelial transformation, and mesothelioma development. For instance, sustained inflammation and fibrosis occur less frequently in IL-1 knockout mice than in wild-type mice (88). In a previous study, the macrophage number and cytokine capacity of IP-10, RANTES, IL-2, and IL-18 were found to be significantly higher in the pleural cavity of MWCNT-treated rats (83). Pro-inflammatory cytokines released by macrophages are exposed to MWCNTs, which then stimulate an amplified release of cytokines from adjacent mesothelial cells (104). The release of IL-1β is dependent on NLRP3 inflammasome activation (105). Moreover, long CNTs can also activate the NLRP3 inflammasome depending on ROS production and cathepsin B activation in macrophages (106). Besides macrophages, lung epithelial cells also contribute to the reprogramming gene expression of mesothelial cells by releasing exosomes after asbestos exposure (107). However, it remains unknown whether lung epithelial cells release exosomes after CNT exposure.

In summary, CNTs are able to transform mesothelial cells primarily *via* ROS and inflammation-mediated mechanisms. DNA damage induced by CNTs has been confirmed both *in vitro* and *in vivo*. CNTs induce DNA damage by directly penetrating the nucleus and disrupting the DNA repair mechanisms. The molecular mechanisms of mesothelioma development related to CNTs are discussed in the next section.

## Gene Expression Profile After CNT Exposure

Specific signaling pathways and related gene expression profiling from normal to precancerous cells seem to be involved in the process of CNT-induced pathological and cellular lesions of mesothelial cells. Generally, MPM induced by asbestos in humans is considered a disease of genetic alterations that involve *CDKN2A*, *NF2*, and *BAP1*, rather than a driver mutation. Genomic mutations and epigenetic alterations seem to be also involved in gene expression modification associated with the transformation of mesothelial cells.

CNTs induce genomic mutations in mesothelial cells in distinct ways. For instance, *p19^Arf^* gene copy number decreases in the region of MWCNT-induced tumors, whereas *p16 ^Ink4a^* does not. In addition, no reduction in *p19^Arf^* gene copy number was observed in mesothelioma induced by asbestos (57).

DNA methylation, which occurs during the early stages of tumorigenesis or in abnormal non-neoplastic tissue, contributes to the inactivation of tumor suppressor genes. Covalent methylation at the C^5^ position of cytosine nucleotides located in cytosine–phosphate–guanine (CpG) islands is involved in tumor development. DNA hypermethylation of CpG islands in *p16^Ink4a^* and *p19^Arf^* (located in exon 1a and the 5’ region flanking exon 1b, respectively) was observed in mesothelial cells in both the tumor and inflammatory areas induced by MWCNT or asbestos (57).

Non-coding RNA alteration is another epigenetic regulation of gene expression, especially for microRNAs (miRNAs). miRNAs are short, non-coding, and double-stranded RNAs with a length of 22 nucleotides that regulate gene expression at the post-translational level by blocking protein translation or inducing mRNA degradation. When exposed to CNTs, miRNAs in mesothelial cells are altered. Previous studies have confirmed that miRNA alterations are involved in the fibrotic responses in wild-type and IL-1 knockout mice after 28 days of MWCNT-7 exposure (88). Studies have also shown that after exposure to MWCNTs for 24 h, miR-155 in human mesothelial cells was upregulated and miR-30 d-5p, miR-34c-5p, miR-28-5p, and miR-324-5p were downregulated (108). Also, miR-155 was overexpressed in the MPM patient tissue (109), suggesting that these miRNAs may be involved in cancer-related signaling pathways. In addition to miRNA, other non-coding RNAs such as long non-coding RNAs and circular RNAs are current popular research topics in oncology. However, there is no evidence to indicate that long non-coding RNA and circular RNA are affected by CNT- or asbestos-induced mesothelioma.

CNTs can disturb signaling pathways, such as the pro-oncogenic pathways and the DNA repair machinery, thereby causing the transformation of mesothelial cells. Long MWCNTs with the same dimensions as asbestos drive MPM development in the same pro-oncogenic signaling pathways, including Src family kinases, Akt, mTOR, ERK1/2, and STAT3, and the disruption of Cdkn2a (57). An *in vitro* study on human mesothelial cells with chronic exposure to CNTs or asbestos demonstrated that both CNT- and asbestos-induced mesothelial cell invasion could result in the upregulation of matrix metalloproteinase-2 (MMP-2) and downregulation of SOD-1 (103). *PLAU*, *STAT3*, *AKT1*, and *VEGFA* are associated with CNT-induced invasion, whereas *CAV1*, *EGFR*, *TIMP2*, and *CCL2* are involved in asbestos-induced invasion. SWCNTs can also induce the transformation of mesothelial cells due to the upregulation of integrin alpha V and cortactin, a downstream target of H-Ras-ERK signaling (110). In addition, inflammatory signaling pathways, including NF-κB and MAPKs, are perturbed by pristine SWCNTs *via* the activation of AP-1, NF-κB, and Akt (99). Animal studies have suggested that p53 can play an important role in the development of CNT-induced mesothelioma (55, 72) and MPM progression in humans (111). However, further investigations on how the molecular signaling pathway is altered during the early stage after asbestos or CNT exposure are required to elucidate the adverse effects of CNT applications.

Although the molecular mechanisms of MPM development induced by CNTs and asbestos show common features, it remains unclear if and how the biopersistent fiber-shaped nanomaterials can induce MPM. Due to the expansive manufacturing and applications of nanomaterials, it is imperative to increase public awareness of the potential risks posed by this rapidly emerging technology (112).

## Hazardous Concerns Regarding Public Health and Occupational Safety

Advances in CNT-based nanotechnology are continually being made; thus, CNTs and their associated products have been used in many industrial and commercial applications. During product manufacturing, transportation, and processing, the release of CNTs into the air poses a major concern in the industry. In addition, the CNTs contained in sports equipment, battery electrodes, and coating may also release hazardous particles to public consumers after abrasion. Furthermore, CNTs have been found in the lung tissue of patients who were exposed to the World Trade Center dust and in the bronchoalveolar fluids of asthmatic Parisian children, suggesting that human tissue can be affected by CNT exposure (113, 114). Based on current investigations, CNTs released from CNT-based products disposed in the soil, sediment, and aquatic ecosystems have shown potential toxicity (115). The effects of improper disposal of such products on aquatic and terrestrial organisms still need to be investigated.

Since environmental and human exposure to CNTs has become a major concern, various government regulations have been published. In 2013, the National Institute for Occupational Safety and Health in the US recommended an exposure limit of 1 μg/m^3^, an 8 h time-weighted average of CNTs (116). Later, the International Agency for Research (IRAC) on Cancer assessed MWCNT-7 as “possibly carcinogenic to humans” (Group 2B), while other types of CNTs were classified as “not classifiable as to their carcinogenicity to humans” (Group 3) in 2014 (117). In 2019, MWCNTs were recommended for carcinogenicity evaluation by the International Agency for Research on Cancer (IARC) Monographs with high priority (118). Recently, CNTs were added to SIN (“Substitute It Now”) list and classified into the “very concerning” level by the Swedish non-profit organization ChemSec due to the carcinogenicity, potential reproductive toxicity, and persistency associated with them (119, 120).

Among 400 fibrous minerals, only six mineral fibers (actinolite, amosite, anthophyllite, chrysotile, crocidolite, and tremolite) are currently under regulation, leaving other materials that may cause asbestos-like disease unregulated (121). According to the International Ban Asbestos Secretariat, 67 countries and regions have set a partial or complete ban on asbestos use (122). Although asbestos has been banned in many countries, it is still extensively used in several developing countries (123). Due to the broad asbestos production and applications in developing countries, researchers have already called for an asbestos ban in China (124). In fact, the prevalence of MPM is expected to peak in the next few decades in China.

Humans can be exposed to CNTs through inhalation or dermal and oral contact in both occupational and non-occupational settings. Little research has been done on the latency period, mesothelioma cell types, and location of CNT-induced malignant proliferation in humans. Therefore, protective measures for occupational exposure should be adopted, and products containing CNTs should be tagged to limit adverse effects. CNTs are considered a largely unrecognized health hazard that needs to be comprehensively assessed. The existing data of asbestos-induced carcinogenic effects may well serve as a guide to prevent CNT-induced diseases.

## Discussion

**Figure 2:**
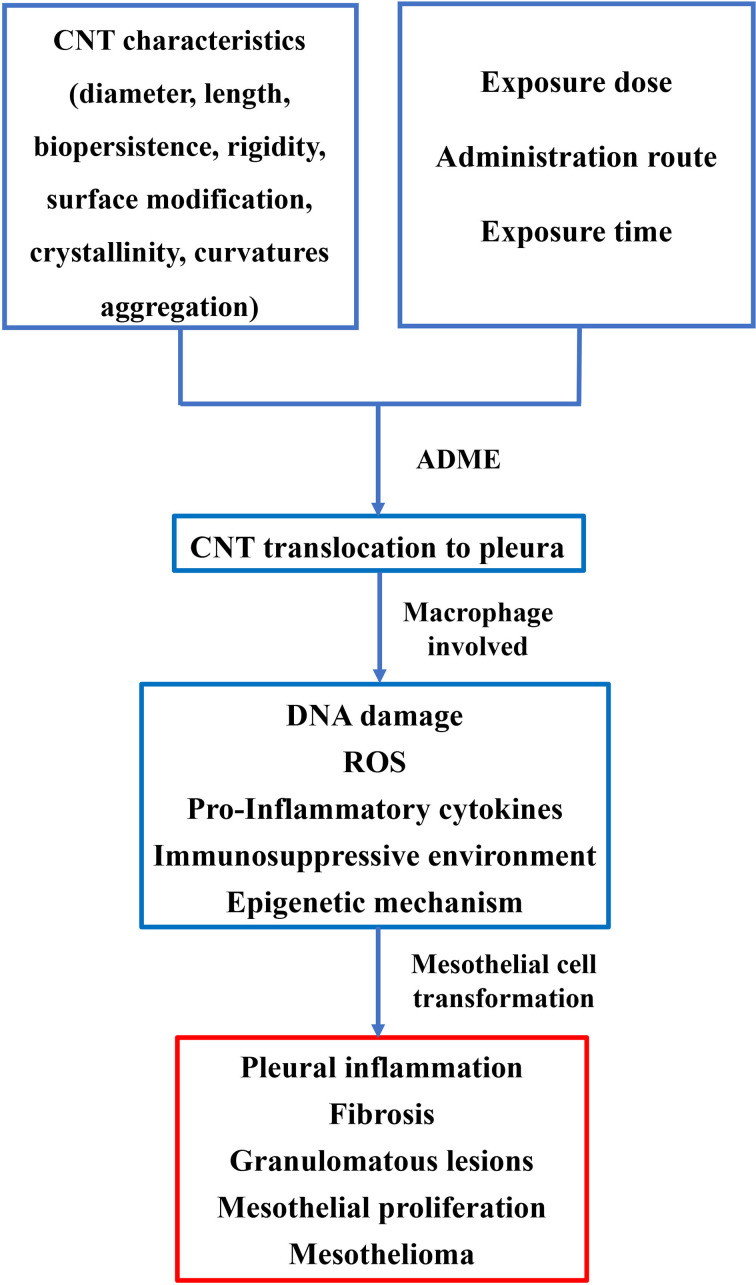
Pathogenesis of MPM induced by CNT exposure. The characteristics of CNT, including length, diameter, surface modification, etc., can influence pathological lesions on pleura. Administrative route, exposure time, and dose also play critical roles in lesion development of the pleura. Macrophages are involved in the process of transformation of mesothelial cells. A hypothesis of DNA damage in mesothelial cells, ROS, pro-inflammatory cytokines, and perturbed signaling pathway may thus be proposed in the transformation of mesothelial cells.

The exceptional physical and chemical properties of CNTs make them one of the most promising candidates for a wide range of applications. However, toxicity induced by nanomaterials has been observed in recent *in vivo* animal studies. The current knowledge of MPM induced by asbestos can facilitate our understanding of CNT-induced mesothelioma growth. MPM development and lung damage related to CNT exposure have not been systematically investigated. Compared to asbestos-related MPM, the development of malignancy by CNTs is more complicated. CNTs are more diverse as a consequence of their unique physicochemical characteristics (**Figure 2**). To better understand CNT-related disease, a comprehensive knowledge base of CNTs is required. Workers who are regularly exposed to CNTs in occupational conditions should be followed up. The recommended exposure limit of CNTs and preventative measures should be formulated by Governmental Occupational Safety and Health Boards to minimize the health risks associated with the use of CNTs. In addition, the environmental release of CNTs should be monitored and regulated by related law, and CNTs should be used under strict supervision, as suggested by the experimental evidence presented in this paper.

## Author Contributions

CZ and LW made all the figures and wrote the manuscript. XZ and MP wrote and revised the manuscript, figures, and table. All authors contributed to the article and approved the submitted version.

## Funding

This work was financially supported by Jinan Clinical Medicine Research Program for Thoracic Cancer (201912007) and a grant from the Special Construction Project Fund for Taishan Mountain Scholars of Shandong Province.

## Conflict of Interest

The authors declare that the research was conducted in the absence of any commercial or financial relationships that could be construed as a potential conflict of interest.

## Publisher’s Note

All claims expressed in this article are solely those of the authors and do not necessarily represent those of their affiliated organizations, or those of the publisher, the editors and the reviewers. Any product that may be evaluated in this article, or claim that may be made by its manufacturer, is not guaranteed or endorsed by the publisher.
