# Supplementary material for: Carbon Nanotubes: A Summary of Beneficial and Dangerous Aspects of an Increasingly Popular Group of Nanomaterials
Source: Front Oncol. 2021 Jul 27;11:693814. doi: 10.3389/fonc.2021.693814 (PMC8353320; doi:10.3389/fonc.2021.693814)
Supplement: Supplementary file 1 [file Table_1.docx]

Supplementary Material

**Supplementary Table 1**. Toxicity studies associated with CNTs induced pathology on pleura

| **CNT** | | **Animal** | **Dose/Max observation period** | | **Major observation** | **Ref.** | |
| --- | --- | --- | --- | --- | --- | --- | --- |
| **Intraperitoneal injection** | | | | | | | |
| MWCNT_tangle1_(D= 14.84±0.50 nm, L^a^=1-5 µm)  MWCNT_tangle2_(D= 10.40±0.32 nm, L^a^=5-20 µm)  MWCNT_long1_ (D= 84.89+1.9 nm, L^a^ mean 13 µm)  MWCNT_long2_ (D= 165.02+4.68 nm, L^a^ Max 56 µm) | | Wide type mice  female C57Bl/6  Age 8 weeks | One single dose of 50 µg/mouse  1 and 7 days | | MWCNT_long1_ and MWCNT_long2_ produce inflammation and granulomas that were similar to inflammatory response caused by long asbestos after 7 days. | (1) | |
| MWCNT-7 (D=100 nm, 27.5% particles≥5µm) | | Male p53 (+/-) mouse  Age 9-11 weeks | One single dose of 1×10^9^ particles (3 mg)/mouse  25 weeks | | The incidence of mesothelioma after MWCNT-7 treated was 87.5% during whole periods of 25 weeks. | (2) | |
| MWCNT-7 (D=70-170 nm, mean 90 nm,  L=1-20 µm mean 2 µm, 27.5% particles ≥5 µm) | | Male p53 (+/-) mice  Age 9–11 weeks | One single dose of  300 µg (1x10^8^ fibers)/mouse  30 µg (1x10^7^ fibers) /mouse  3 µg (1x10^6^ fibers)/mouse  1 year | | Cumulative incidence of peritoneal mesothelioma induced by MWCNT-7 displayed dose-dependent manner. | (3) | |
| MWCNT50a (MNCNT-7)  (D=49.95 ± 0.63 nm, L=5.29 ± 0.12 µm)  MWCNT50b  (D=52.4 ± 0.72 nm, L=4.6 ± 0.10 µm)  MWCNT115  (D= 116.2 ± 1.6 nm, L=4.88 ± 0.10 µm)  MWCNT145  (D= 143.5 ± 1.6 nm, L= 4.34 ± 0.08 µm)  MWCNTtngl (D^a^=15 nm, L^a^=3 µm) | | Male and female rats  Age 6 weeks | Total dose of 1 mg or 10 mg/rat  1 year | | MWCNT-7 induced severe chronic inflammation and mesothelioma development because of its thin diameter and high crystallinity. | (4) | |
| MWCNT-M (D mean 66.8 nm, L mean 6.65 µm) (MWCNT-7)  MWCNT-N (D mean 59.2 nm, L mean 5.48 µm)  MWCNT-WL (D mean 70.9 nm, L mean 7.31 µm)  MWCNT-SD1 (D mean 177.4 nm, L mean 4.51 µm)  MWCNT-WS (D mean 44.5 nm, L^a^=0.5-2 µm)  MWCNT-SD2 (D mean 13.5 nm, L^a^=3 µm)  MWCNT-T (D mean 35.8 nm, L^a^=0.732 µm) | | Male Fischer 344 rats  Age 10 weeks | a single dose of 1 mg/kg  1 year | | Four types of MWCNTs, such as M, N, WL, and SD1, which have straight, acicular in shape and few agglomerates, induced nearly 100% incidences of mesothelioma development. | (5) | |
| MWCNT A^b^ (D=85±1.6 nm, L=8.57±1.51µm)  MWCNT B^b^ (D=62±1.71 nm, L=9.3±1.63 µm)  MWCNT C^b^ (D=40±1.57 nm, L=10.24±1.64 µm)  MWCNT D^b^ (D=37±1.45 nm, L=7.91±1.4 µm) | | Male Wistar Han rats | a single dose of 1×10^9^particles^b^/rat or  5×10^9^particles^b^/rat  2 years | | Highest frequencies and earliest appearances of mesothelioma development was observed after treatment with the rather straight MWCNT types A and B. | (6) | |
| Pristine MWNTs:NTlong  (L mean 10.7±1.4 µm)  alkyl functionalized MWNTs: NT-Alkyl  (L mean 8.1±0.6 µm)  tri(ethylene glycol) functionalized MWNTs: NT-TEG  (L mean 2.4±0.2 µm) | | C57BL6 mice  Age 6-8 weeks | One single dose of 50 µg/mouse  1 and 7 days | | Hydrophilic surface modification leading to shorten or untangling/debundling of aqueous dispersions of functionalized-MWNTs will help to resolve toxicological risks associated with long-fiber exposure. | (7) | |
| **Intrascrotal injection** | | | | | | | |
| MWCNT-7  (D=70-100 nm of 82% particles,  L=70-110 µm 72.5 % particles) | | Male Fischer rats  Age 12 weeks | a single dose of 0.24 mg/rat  1 year | | MWCNT-7 induced intraperitoneally disseminated mesothelioma in the incidence of 85.7% after 52 weeks. | (8) | |
| **Inhalation exposure** | | | | | | | |
| MWCNTs  (D=30-50 nm, L=0.3-50 µm) | Male C57BL6 mice  Age 6-8 weeks | | Low dose (0.2 mg/kg) or high dose (4 mg/kg) for 6h  1 day, 2 weeks, 6 weeks or 14 weeks | MWCNTs were embedded in the subpleural wall, while increasing of subpleural fibrosis was observed after 2 and 6 weeks after inhalation. | | (9) |  |
| MWCNT-7**^c^**  (D=92.9-98.2 nm, L=5.4-5.9 µm) | Male and female F344 rats  Age 6 weeks | | 0.02, 0.2, and 2 mg/m^3^ for 6 h/day, 5 days/week for 104 weeks  2 years | Incidence of simple mesothelial hyperplasia of the parietal pleural and focal fibrosis of the parietal pleura side of the diaphragm was observed in male rats exposed to the high dose. | | (10) |  |
| MWCNT-7  (D^d^=1.59 µm, L ND) | Male B6C3F1 mice  Age 6 weeks | | 5 mg/m^3^, 5 hours/day, 5 days/week, for 15 days  17 months | Only 9% mice exposed MWCNT and methylcholanthrene developed mesothelioma, whereas mice administered MWCNT did not develop mesothelioma. | | (11) |  |

| **Pharyngeal respiration** | | | | | |
| --- | --- | --- | --- | --- | --- |
| MWCNT-7  D=49±13.4 nm, L mean 3.86 µm | Male C57BL/6J mice  Age 7 weeks | One single dose of 10, 20, 40, 80 µg/mouse  1day, 1 week, 4 weeks, 8 weeks | After1 day exposure at dose of 80 µg/mouse, 0.6% MWCNT lung burden was in the subpleural regions. At day 56 approximately 1 in every 400 penetrated MWCNTs was in either the subpleural tissue or intrapleural space. | (12) |  |
| MWCNT_short_  (D=25.7±1.6 nm, L=1-2 µm)  MWCNT_tangle_  (D=14.84±0.05 nm, L=1-5 µm)  MWCNT_long_  (D=165.02±4.68 nm, L mean 36 µm, 84.26% fiber great than 15 µm) | Female C57BL/6 mice  Age 8-12 week | One single dose of 50 µg/mouse  1 and 6 weeks | Long MWCNT were retained at the parietal pleura and caused inflammation and lesion development. | (13) |  |
| **Intratracheal instillation** | | | | |  |
| SWCNTs  (D=1.7-2.1 nm, L=0.05-8.14 µm, mean 0.5 µm)  MWCNT-7  (D=60-100 nm, L=0.12-21.5 µm, mean 1.81 µm) | Male Wistar rat  Age 9 weeks | One single dose of 0.15 mg/kg or 1.5 mg/kg  1, 3, 7, 30, 90 days | MWCNT-7 induced greater levels of pleural inflammation than did short SWCNTs. | (14) |  |
| **Intratracheal intrapulmonary spraying** | | | | |  |
| MWCNT-N  (D ND, L=3.64 ± 2.26 µm, mean 3.02 µm)  MWCNT-7  (D ND, L= 5.11 ± 2.91 µm, mean 4.47 µm) | Male Fisher 344 rats  Age 10 weeks | 1.25 mg/rat (Five doses at 0.25 mg/rat, five times over 9 days period)  Six hours after the last exposure | MWCNTs treatment caused visceral mesothelial cell proliferation and inflammation in the pleural cavity. | (15) |  |
| MWCNT-L (D mean 150 nm, L mean 8 µm)  MWCNT-S (D mean 15 nm, L mean 3 µm) | Male F344 rats  Age 8 weeks | 1.625 mg⁄ rat (13 doses at 0.125 mg/rat, 13 times over 24 weeks period)  24 hours after the last exposure | MWCNT-L induced stronger inflammatory reactions in the pleural cavity and fibrosis and patchy parietal mesothelial proliferation lesions. | (16) |  |
| MWCNT 1  (L=7.41±3.52 µm, D^a^=30-200, mean 177 nm)  MWCNT 2  (L=4.27±2.88 µm, D^a^=70-170 nm, mean 90 nm) | Male F344 rats  Age 8 weeks | 2 mg/rat (8 doses at 0.25 mg/rat, 2 times/week for 4 weeks)  24 h and at 3 months after the last exposure | MWCNTs induced pleural inflammation, persistent pleural fibrosis, and mesothelial proliferation in both the visceral and parietal pleura. | (17) |  |
| MWCNT-N (D^a^=1-20 nm)  Unfiltered (L=4.2±2.9 µm)  Flow-through (L=2.6±1.6 µm)  Retained (>2.6 µm) | Male F344 rats  Age 10 weeks | 1 mg⁄rat (8 doses at 0.125 mg/rat, 8 times over 2 weeks period)  109 weeks after the last exposure | Both the unfiltered and flow-through fractions induced mesothelioma. | (18) |  |
| MWCT-7  (ND) | Male F344 rats  Age 10 weeks | 1.5 mg/rat (12 doses at 0.125 mg/rat, once a week for 12 weeks)  2 years after the last exposure | The incidence of malignant mesothelioma in the MWCNT‐7 group was significantly higher than in the vehicle control group. | (19) |  |
| **Intrapleural injection** | | | | |  |
| MWCNT_Straight_  SNT (D=125 nm, L <15 µm)  MWCNT_Long, straight_  LNT (D=165 nm, L 85% particles >15 µm) | Female C57BL/6 mice  Age 8 weeks | 1 and 12 weeks exposure:  One single dose of 5 µg/mouse of SNT and LNT  6 months and 1year exposure:  One single dose of 2.5 µg/mouse of LNT  Up to 20 months:  One single dose of 1, 0.5, 0.2 µg/mouse of LNT | CNT induced mesothelioma exhibits similar common key pro-oncogenic molecular events compared with asbestos throughout the latency period of disease progression. | (20) |  |
| MWCNTshort (D**^a^**=20-30 nm, L**^a^**=0.5-2 µm)  MWCNTtangle1 (D=14.84±0.05 nm, L**^a^**=1-5 µm)  MWCNTtangle2 (D=10.4±0.32 nm, L**^a^**=5-20 µm)  MWCNTlong1 (D=84.89±1.9 nm, L**^a^** mean 13 µm)  MWCNTlong2 (D=165.02±4.68 nm, L**^a^** max13 µm) | Female C57Bl/6 mice  Age 8 weeks | One single dose of 5 µg/mouse  1 day, 1 week, 4 weeks, 12 weeks and 24 weeks | Long CNT retained in pleural cavity and induced acute inflammation and progressive fibrosis on the parietal pleura. | (21) |  |
| MWCNT1 MWCNT-7  (D percentile 70 nm, L percentile 5.3 µm)  MWCNT2 (D percentile 31 nm, L percentile 0.843 µm) | Wide type C57BL/6 mice  IL1α/β knockout mice | One single dose of 50 µg/mouse  and 100 µg/mouse  4 weeks | MWCNT1 (MWCNT-7) induces more severe inflammatory responses than MWCNT2 and asbestos in WT mice. WT mice were more prone to development of sustained inflammation and fibrosis than IL1-KO mice. | (22) |  |
| Silver Nanowire  AgNW_3_ (D=115±3 nm, L mean 3 µm)  AgNW_5_ (D=118±3 nm, L mean 5 µm)  AgNW_10_ (D=128±2 nm, L mean 10 µm)  AgNW_14_ (D=121±3 nm, L mean 14 µm)  AgNW_28_ (D=120±4 nm, L mean 28 µm) | Female C57Bl/6 mice  Age 9 weeks | One single dose of  5 µg/mouse  1 day and 1 week | Fibers beyond 5 μm in length are pathogenic to the pleura. | (23) |  |
| Carbon nanofibers CNF_tangle1_  (D=24.79 ± 0.4 nm, L ND, D_b_<0.66, SBPL<0.87)  MWCNT_tangle1_  (L=16.37 ± 0.2 nm, L ND, D_b_<0.66, SBPL<0.87)  MWCNT_tangle2_  (D=15.64 ± 0.1 nm, L ND, D_b_<0.66, SBPL<0.87)  MWCNT_tangle3_  (D=7.75 ± 0.1 nm, L ND, D_b_<0.66, SBPL<0.87)  MWCNT_tangle4_  (D=16.7 ± 0.2 nm, L ND, D_b_<0.66, SBPL<0.87)  MWCNT_long1_  (D=58.3 ± 1.0 nm, L=10.02 ± 0.3 µm, D_b_>0.97, SBPL>1.09) | Female ICR mice  Age 6 weeks | One single dose  1 day exposure: 1, 2.5, 5 µg/mouse  4 weeks exposure:  5 µg/mouse | A bending ratio of 0.97 and a static bending persistence length of 1.08 are the threshold rigidity values for asbestos-like pathogenicity. | (24) |  |

ND not determined, D_b_ bending ratio, SBPL static bending persistence length

a Values obtained from the manufacturer.

^b^ WHO fibers, a fiber length of at least 5 μm and a fiber diameter of less than 3 μm with an aspect ratio (ratio of fiber length to fiber diameter) of at least 3:1.

^c^ Fibers collected from the inhalation chamber.

^d^Mass median aerodynamic diameter

**References**

1. Poland CA, Duffin R, Kinloch I, Maynard A, Wallace WAH, Seaton A, et al. Carbon nanotubes introduced into the abdominal cavity of mice show asbestos-like pathogenicity in a pilot study. *Nature Nanotechnology* (2008) 3(7):423-8. doi: 10.1038/nnano.2008.111. PubMed PMID: WOS:000257984700015.

2. Takagi A, Hirose A, Nishimura T, Fukumori N, Ogata A, Ohashi N, et al. Induction of mesothelioma in p53+/- mouse by intraperitoneal application of multi-wall carbon nanotube. *Journal of Toxicological Sciences* (2008) 33(1):105-16. doi: 10.2131/jts.33.105. PubMed PMID: WOS:000273413000011.

3. Takagi A, Hirose A, Futakuchi M, Tsuda H, Kanno J. Dose-dependent mesothelioma induction by intraperitoneal administration of multi-wall carbon nanotubes in p53 heterozygous mice. *Cancer Science* (2012) 103(8):1440-4. doi: 10.1111/j.1349-7006.2012.02318.x. PubMed PMID: WOS:000306901900001.

4. Nagai H, Okazaki Y, Chew SH, Misawa N, Yamashita Y, Akatsuka S, et al. Diameter and rigidity of multiwalled carbon nanotubes are critical factors in mesothelial injury and carcinogenesis. *Proceedings of the National Academy of Sciences of the United States of America* (2011) 108(49):E1330-E8. doi: 10.1073/pnas.1110013108. PubMed PMID: WOS:000297683800010.

5. Sakamoto Y, Hojo M, Kosugi Y, Watanabe K, Hirose A, Inomata A, et al. Comparative study for carcinogenicity of 7 different multi-wall carbon nanotubes with different physicochemical characteristics by a single intraperitoneal injection in male Fischer 344 rats. *Journal of Toxicological Sciences* (2018) 43(10-12):587-600. doi: 10.2131/jts.43.587. PubMed PMID: WOS:000457816700003.

6. Rittinghausen S, Hackbarth A, Creutzenberg O, Ernst H, Heinrich U, Leonhardt A, et al. The carcinogenic effect of various multi-walled carbon nanotubes (MWCNTs) after intraperitoneal injection in rats. *Particle and Fibre Toxicology* (2014) 11. doi: 10.1186/s12989-014-0059-z. PubMed PMID: WOS:000345873300001.

7. Ali-Boucetta H, Nunes A, Sainz R, Herrero MA, Tian B, Prato M, et al. Asbestos-like Pathogenicity of Long Carbon Nanotubes Alleviated by Chemical Functionalization. *Angewandte Chemie-International Edition* (2013) 52(8):2274-8. doi: 10.1002/anie.201207664. PubMed PMID: WOS:000314998500027.

8. Sakamoto Y, Nakae D, Fukumori N, Tayama K, Maekawa A, Imai K, et al. Induction of mesothelioma by a single intrascrotal administration of multi-wall carbon nanotube in intact male Fischer 344 rats. *Journal of Toxicological Sciences* (2009) 34(1):65-76. doi: 10.2131/jts.34.65. PubMed PMID: WOS:000263149200006.

9. Ryman-Rasmussen JP, Cesta MF, Brody AR, Shipley-Phillips JK, Everitt JI, Tewksbury EW, et al. Inhaled carbon nanotubes reach the subpleural tissue in mice. *Nature Nanotechnology* (2009) 4(11):747-51. doi: 10.1038/nnano.2009.305. PubMed PMID: WOS:000272413500017.

10. Kasai T, Umeda Y, Ohnishi M, Mine T, Kondo H, Takeuchi T, et al. Lung carcinogenicity of inhaled multi-walled carbon nanotube in rats. *Particle and Fibre Toxicology* (2016) 13. doi: 10.1186/s12989-016-0164-2. PubMed PMID: WOS:000385294400001.

11. Sargent LM, Porter DW, Staska LM, Hubbs AF, Lowry DT, Battelli L, et al. Promotion of lung adenocarcinoma following inhalation exposure to multi-walled carbon nanotubes. *Particle and Fibre Toxicology* (2014) 11. doi: 10.1186/1743-8977-11-3. PubMed PMID: WOS:000330053200001.

12. Mercer RR, Hubbs AF, Scabilloni JF, Wang L, Battelli LA, Schwegler-Berry D, et al. Distribution and persistence of pleural penetrations by multi-walled carbon nanotubes. *Particle and Fibre Toxicology* (2010) 7. doi: 10.1186/1743-8977-7-28. PubMed PMID: WOS:000282982700001.

13. Murphy FA, Poland CA, Duffin R, Donaldson K. Length-dependent pleural inflammation and parietal pleural responses after deposition of carbon nanotubes in the pulmonary airspaces of mice. *Nanotoxicology* (2013) 7(6):1157-67. doi: 10.3109/17435390.2012.713527. PubMed PMID: WOS:000322836800011.

14. Fujita K, Fukuda M, Endoh S, Maru J, Kato H, Nakamura A, et al. Pulmonary and pleural inflammation after intratracheal instillation of short single-walled and multi-walled carbon nanotubes. *Toxicology Letters* (2016) 257:23-37. doi: 10.1016/j.toxlet.2016.05.025. PubMed PMID: WOS:000379653300003.

15. Xu J, Futakuchi M, Shimizu H, Alexander DB, Yanagihara K, Fukamachi K, et al. Multi-walled carbon nanotubes translocate into the pleural cavity and induce visceral mesothelial proliferation in rats. *Cancer Science* (2012) 103(12):2045-50. doi: 10.1111/cas.12005. PubMed PMID: WOS:000312032100002.

16. Xu J, Alexander DB, Futakuchi M, Numano T, Fukamachi K, Suzui M, et al. Size- and shape-dependent pleural translocation, deposition, fibrogenesis, and mesothelial proliferation by multiwalled carbon nanotubes. *Cancer Science* (2014) 105(7):763-9. doi: 10.1111/cas.12437. PubMed PMID: WOS:000340602800003.

17. Liao D, Wang Q, He J, Alexander DB, Abdelgied M, El-Gazzar AM, et al. Persistent Pleural Lesions and Inflammation by Pulmonary Exposure of Multiwalled Carbon Nanotubes. *Chemical Research in Toxicology* (2018) 31(10):1025-31. doi: 10.1021/acs.chemrestox.8b00067. PubMed PMID: WOS:000447680300006.

18. Suzui M, Futakuchi M, Fukamachi K, Numano T, Abdelgied M, Takahashi S, et al. Multiwalled carbon nanotubes intratracheally instilled into the rat lung induce development of pleural malignant mesothelioma and lung tumors. *Cancer Science* (2016) 107(7):924-35. doi: 10.1111/cas.12954. PubMed PMID: WOS:000384724100008.

19. Numano T, Higuchi H, Alexander DB, Alexander WT, Abdelgied M, El-Gazzar AM, et al. MWCNT-7 administered to the lung by intratracheal instillation induces development of pleural mesothelioma in F344 rats. *Cancer Science* (2019) 110(8):2485-92. doi: 10.1111/cas.14121. PubMed PMID: WOS:000478328500001.

20. Chernova T, Murphy FA, Galavotti S, Sun XM, Powley IR, Grosso S, et al. Long-Fiber Carbon Nanotubes Replicate Asbestos-Induced Mesothelioma with Disruption of the Tumor Suppressor Gene Cdkn2a (Ink4a/Arf). *Current Biology* (2017) 27(21):3302-+. doi: 10.1016/j.cub.2017.09.007. PubMed PMID: WOS:000414581700022.

21. Murphy FA, Poland CA, Duffin R, Al-Jamal KT, Ali-Boucetta H, Nunes A, et al. Length-Dependent Retention of Carbon Nanotubes in the Pleural Space of Mice Initiates Sustained Inflammation and Progressive Fibrosis on the Parietal Pleura. *American Journal of Pathology* (2011) 178(6):2587-600. doi: 10.1016/j.ajpath.2011.02.040. PubMed PMID: WOS:000298306900015.

22. Arnoldussen YJ, Skaug V, Aleksandersen M, Ropstad E, Anmarkrud KH, Einarsdottir E, et al. Inflammation in the pleural cavity following injection of multi-walled carbon nanotubes is dependent on their characteristics and the presence of IL-1 genes. *Nanotoxicology* (2018) 12(6):522-38. doi: 10.1080/17435390.2018.1465139. PubMed PMID: WOS:000439981600003.

23. Schinwald A, Murphy FA, Prina-Mello A, Poland CA, Byrne F, Movia D, et al. The Threshold Length for Fiber-Induced Acute Pleural Inflammation: Shedding Light on the Early Events in Asbestos-Induced Mesothelioma. *Toxicological Sciences* (2012) 128(2):461-70. doi: 10.1093/toxsci/kfs171. PubMed PMID: WOS:000307698500015.

24. Lee D-K, Jeon S, Han Y, Kim S-H, Lee S, Yu IJ, et al. Threshold Rigidity Values for the Asbestos-like Pathogenicity of High-Aspect-Ratio Carbon Nanotubes in a Mouse Pleural Inflammation Model. *Acs Nano* (2018) 12(11):10867-79. doi: 10.1021/acsnano.8b03604. PubMed PMID: WOS:000451789200026.
